# Fiberoptic endoscopic evaluation of swallowing in German stroke units: a retrospective diagnosis-related group analysis (2019–2024)

**DOI:** 10.1186/s42466-026-00506-3

**Published:** 2026-06-11

**Authors:** Matthias N. Ungerer, Dirk Bartig, Annika Moreno Agredo, Tobias Braun, Christoph Gumbinger

**Affiliations:** 1 Department of Neurology, Heidelberg University Hospital, 69120 Heidelberg, Germany; 2 Department of Neurology, Kreiskrankenhaus Bergstraße, 64646 Heppenheim, Germany; 3 DRG-Market, 49082 Osnabrück, Germany; 4 Department of Neurology, Lahn-Dill-Kliniken Wetzlar, 35578 Wetzlar, Germany; 5 Department of Neurology, Justus-Liebig-University, 35392 Giessen, Germany

**Keywords:** Dysphagia, Stroke unit, Acute stroke

## Abstract

**Background:**

Fiberoptic Endoscopic Evaluation of Swallowing (FEES) is a safe and effective tool for post-stroke dysphagia assessment. The German Stroke Organization implemented FEES quality benchmarks for stroke unit certification in 2025. This study establishes baseline data on FEES implementation across German stroke units.

**Methods:**

A retrospective analysis of national diagnosis-related group data (2019–2024) was performed. We analyzed data on cases with stroke unit treatment, examining coding of FEES, dysphagia, and PEG placement, and their relationships with stroke type, institutional service level, and patient demographics.

**Results:**

A total of 1,540,947 cases with stroke unit treatment were analyzed across the six-year study period. FEES procedures were coded in 2.7% of cases, increasing from 2.4% (2019) to 3.1% (2024). FEES coding was more common in cases with intracerebral hemorrhage (5.7%) and ischemic stroke (3.4%). Dysphagia was coded in 13.3% of cases, with higher rates in intracerebral hemorrhage (28.5%) compared to ischemic stroke (16.4%). Cases treated in comprehensive stroke units coded FEES procedures (3.6%) more frequently than regional (2.5%) and basic stroke units (1.5%). Sex-based disparities emerged: cases with males were coded with FEES more often (3.1%) than females (2.4%), despite dysphagia being documented more frequently among cases with females (males 13.0%, females 13.8%). The proportion of cases with documented dysphagia among those coded with FEES was 76.2% and remained stable throughout the study period, suggesting broadly consistent case selection.

**Conclusion:**

Documentation of FEES among cases with stroke unit treatment has increased but remains substantially below the dysphagia documentation rates, suggesting room for broader implementation. Sex-based disparities in FEES documentation need further investigation. Institutional factors impact documentation rates, indicating a need for tailored quality benchmarks by stroke service level. Improving reimbursement, staff training, and streamlined workflows could enhance access to FEES in German stroke units.

## Introduction

Dysphagia represents a highly prevalent complication after an acute stroke, affecting an estimated 50 to 80% of patients within the immediate post-stroke period [1, 2]. The condition is strongly associated with severe and often devastating post-stroke complications, including aspiration pneumonia, malnutrition, dehydration, prolonged hospitalization, and elevated mortality rates [3]. Multiple studies have identified several risk factors for post-stroke dysphagia (PSD), including hypertension, advanced age, and stroke severity [4, 5]. Current guidelines mandate the implementation of standardized dysphagia screening protocols for all stroke patients as an immediate priority upon hospital admission [5]. When clinical bedside screening examinations prove inconclusive, additional diagnostic procedures become necessary. Fiberoptic Endoscopic Evaluation of Swallowing (FEES), an endoscopic examination technique permitting direct visualization of the pharyngeal structures and swallowing mechanism, provides a definitive diagnosis of dysphagia and enables precise identification of the underlying pathomechanism and aspiration risk [6, 7]. Instrumental diagnostics are the only means to detect silent aspirations, which, by definition, evade clinical screening examinations. FEES procedures improve clinical decision-making by providing objective evidence of swallowing efficiency. This is associated with reduced aspiration pneumonia, informs the need for enteral feeding or tube placement, and leads to changes in oral diets [8]. Beyond immediate clinical consequences, untreated or inadequately managed PSD contributes substantially to prolonged hospital stays and escalating healthcare costs, underscoring the critical importance of accurate diagnosis and comprehensive treatment [9–11]. Recent findings have shown that utilization of FEES in the outpatient sector of the German healthcare system remains underdeveloped due to the lack of reimbursement and limited availability, despite its significance in treating dysphagia [12]. 

Germany established a comprehensive and standardized FEES Curriculum in 2014, designed to ensure consistency in training and to establish quality criteria and safety standards for the execution of FEES procedures [13]. A comprehensive survey of 190 German institutions demonstrated that more than 40% had initiated FEES investigations only after the curriculum was implemented, suggesting an important impact of the training program [14]. A multicenter FEES registry study conducted over three years across 23 hospitals in Germany and Switzerland enrolled 2,401 patients. The study confirmed the procedure’s safety in stroke patients, with complications occurring in less than 2% of cases, all of which resolved spontaneously. Additionally, FEES led to significant therapeutic changes in over 50% of cases, primarily involving advancements in the functional oral diet [15]. Standardizing integrated FEES reports has been proposed to streamline documentation and enhance interpretability [16]. These developments have collectively led to an increased use of FEES in Germany. However, most current knowledge regarding the utilization of FEES in clinical practice comes from survey-based methodologies rather than robust administrative healthcare datasets. Recent studies have further highlighted the potential underutilization of FEES in specific patient populations. For instance, one study found that among patients undergoing mechanical thrombectomy (MT), approximately 80% showed dysphagia confirmed by FEES. In many cases, the standard dysphagia screening protocols were found to be significantly less effective than FEES in detecting dysphagia [2]. Accurate information on the use of FEES is essential, particularly as the German Stroke Society (DSG) revised its certification criteria for stroke units to include FEES procedures, starting in 2025. Comprehensive stroke units (cSUs) will be required to conduct at least 35 FEES procedures annually, with a recommended target of 50. Discussions are ongoing regarding requirements for regional and basic stroke units. This development underscores the growing consensus that FEES should be standard practice in all certified stroke units [17]. 

The primary aim of this study was to establish comprehensive, evidence-based baseline data characterizing the nationwide implementation of FEES procedures in acute stroke patients managed within German stroke units. We analyzed temporal trends using diagnosis-related group (DRG) data from 2019 to 2024, examined variations in practice patterns across different stroke types and institutional stroke service levels, and investigated potential sex-based disparities in FEES utilization.

## Methods

### Study design and data source

We conducted a retrospective cohort analysis utilizing encrypted, anonymized case-level data derived from the national DRG database maintained by the “*Institut für das Entgeltsystem im Krankenhaus”* (InEK) [18]. All acute-care hospitals providing inpatient services throughout Germany are legally mandated by the Hospital Reimbursement Act (Krankenhausentgeltgesetz, § 21 KHEntgG) to submit comprehensive case-level data to InEK within defined timeframes. InEK performs systematic, multi-level data quality assurance through automated data format validation and plausibility checks to identify and flag potential coding errors. This study did not collect any additional data. Institutional ethics approval was not required for this secondary analysis of anonymized administrative data.

### Study population and case selection

We included all cases treated in German hospitals between 2019 and 2024 with documented stroke unit (SU) treatment irrespective of their primary hospital diagnosis. SU treatment was identified using the German “*Operationen- und Prozeduren-Schlüssel”* (OPS) coding system, specifically the OPS codes 8-981.- and 8-98b.-, which denotes stroke unit admission and care, as has been done previously [19]. These OPS codes represent stratified stroke service levels reflecting institutional capabilities and quality standards:

- OPS 8-98b: Basic stroke units, typically in smaller institutions, providing 24/7 access to qualified physicians capable of administering intravenous thrombolysis, with or without teleneurological consultation capabilities and access to cerebral vascular imaging; generally does not include in-house MT capability.
- OPS 8-981.- (before 2021): Comprehensive stroke units providing 24/7 in-house neurologist coverage, meeting defined quality benchmarks, and offering access to MT services.
- OPS 8-981.2- (introduced 2021): Regional stroke units, providing 24/7 neurologist coverage and quality benchmarks but lacking in-house MT capability; requiring inter-hospital transfer for thrombectomy procedures.
- OPS 8-981.3- (introduced 2021): Comprehensive stroke units, providing 24/7 neurologist coverage, quality benchmarks, and in-house MT capability.

Applicable primary hospital diagnoses included transient ischemic attack (TIA), acute ischemic stroke (IS; ICD-10 I63.-), stroke not specified as ischemic or hemorrhagic (ICD-10 I64.-) and acute hemorrhagic stroke comprising intracerebral hemorrhage (ICH; ICD-10 I61.-), subarachnoid hemorrhage (I60.-), and other non-traumatic intracranial hemorrhage (I62.-).

### Primary variables and outcome measures

We extracted and analyzed data on the following key variables:

- FEES procedures: Identified by OPS code 1-613.
- MT procedures: Identified by OPS code 8-836.80.
- Dysphagia: Identified as secondary diagnosis ICD-10 code R13.-.
- Percutaneous Endoscopic Gastrostomy (PEG): Identified by OPS code 5-431.2-.
- Stroke type: Classified by primary diagnosis ICD-10 codes (I63.- for ischemic stroke; I61.- for intracerebral hemorrhage).
- Patient demographics: Sex (male/female) and age category (younger than 80 years versus 80 years and older).

Coding dysphagia as a secondary diagnosis indicates dysphagia requiring treatment that relates to the primary hospital diagnosis. While this does not establish a direct causal link to a stroke diagnosis, we assumed that in most of these cases, the coding of dysphagia corresponded with PSD. PEG placement was used as a surrogate for severe dysphagia. Comprehensive subgroup analyses examined specific stroke types (ischemic versus hemorrhagic) and stratified results by stroke service level and patient sex to identify potential institutional and demographic patterns. We calculated the proportion of cases coded for both dysphagia and FEES to all cases coded with dysphagia to estimate the implementation of FEES in appropriate cases. The proportion of cases coded with both dysphagia and FEES to all cases with FEES coding was used to verify consistent case selection.

### Statistical analysis and data management

All case-level data were analyzed using standard descriptive statistics, including frequencies, percentages, and proportions. To enhance interpretability, we calculated the proportion of males assigned a specific code (such as FEES or dysphagia) alongside the proportion of males in the group who did not receive that specific code. This provided an additional comparator for sex-based analyses. Categorical comparisons between groups were performed using chi-square (χ²) statistical tests. Statistical significance was defined a priori as a two-tailed probability value (p-value) of 0.05 or less. Percentages were calculated using full underlying values and are generally reported to one decimal place in the text and two decimal places in the tables. Reported rates were calculated as proportions of cases with the respective code among all cases in the relevant group or subgroup. All statistical analyses and data visualization were performed using IBM SPSS^®^ Statistics software, Version 29 (IBM Corporation, Armonk, NY, USA).

## Results

### Overall cohort characteristics and FEES documentation trends

We analyzed 1,540,947 individual patient cases with stroke unit care treated in German hospitals during the six-year study period from 2019 to 2024 (Table 1). Dysphagia was documented as a secondary diagnosis in 205,602 cases, representing 13.3% of the overall cohort. FEES procedures (identified by OPS code 1-613) were coded in 41,916 cases, representing 2.7% of all cases with SU care. Notably, FEES documentation showed a consistent upward trend throughout the observation period, rising from 2.4% of cases in 2019 to 3.1% in 2024, a 32% relative increase. Among patients who underwent FEES examination, the majority were male (58.9%), and 43.1% were aged 80 years or older. Coding for PEG placement, which was seen as a surrogate for severe dysphagia, was observed in 1.4% of all SU cases and showed no significant trend. Both the absolute number and the relative proportion of cases with documentation of at least one FEES procedure increased consistently throughout the study period. In contrast, the proportion of cases with secondary diagnosis coding for dysphagia remained relatively stable across the six years. The proportion of cases with documented FEES among cases with documented dysphagia increased consistently, rising from 13.1% in 2019 to 19.2% in 2024 (mean 15.5%). Notably, despite the substantial increase in coded FEES procedures, the proportion of cases with documented dysphagia among those with documented FEES remained remarkably stable throughout the observation period, with a mean of 76.2%, suggesting broadly consistent case selection.

**Fig. 1:**
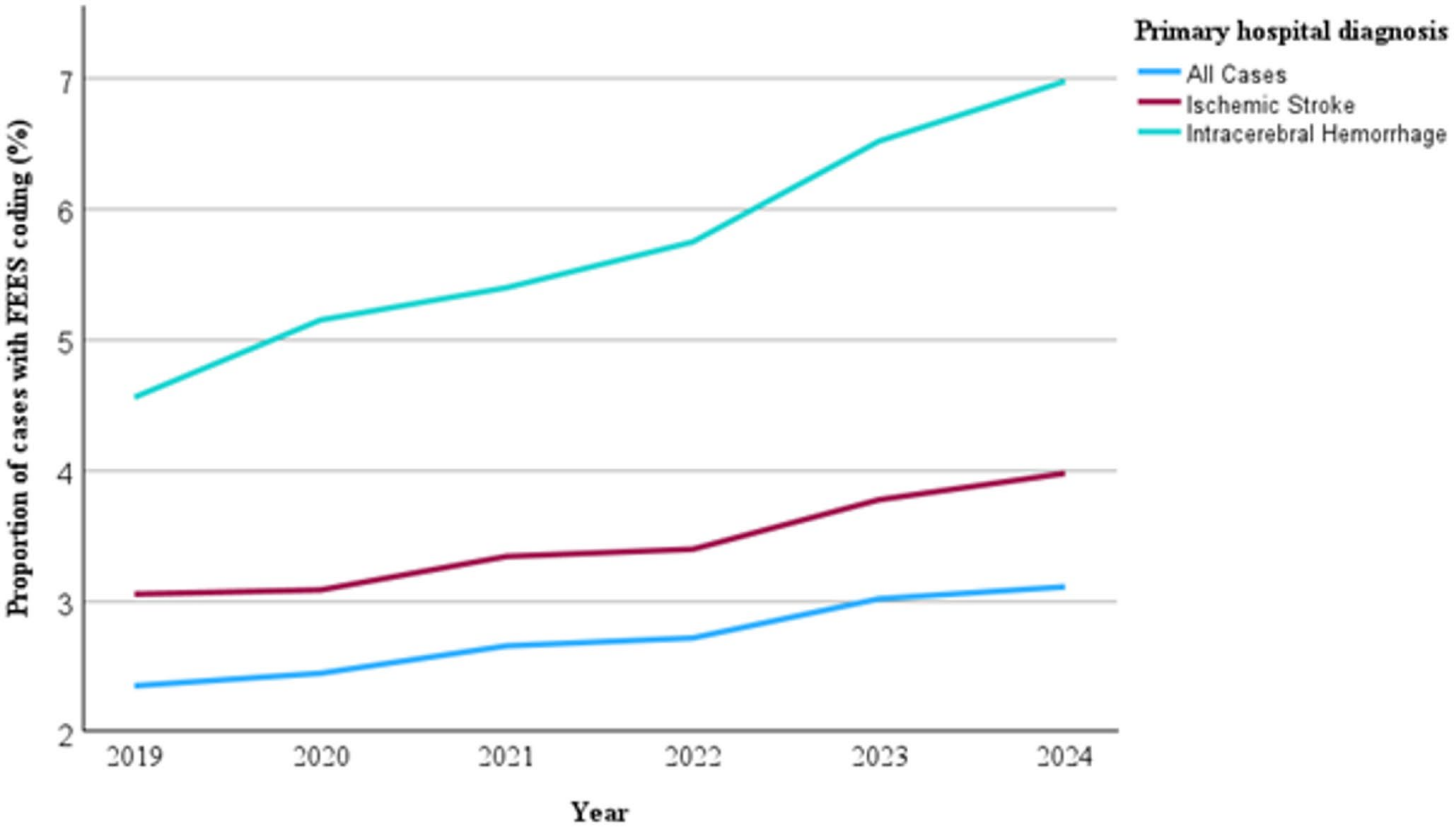
Proportion of  cases with FEES coding relative to all SU cases by primary hospital diagnosis over time FEES, Fiberoptic Endoscopic Evaluation of Swallowing. SU, Stroke unit

**Table 1:** Documented dysphagia, FEES, and selected case characteristics among stroke unit cases in Germany, 2019–2024.

		All SU cases	SU cases with dysphagia	SU cases with PEG placement	SU cases with FEES	SU cases with both FEES and Dysphagia	SU cases with FEES (male)	SU cases with FEES (≥ 80years)
2019	Cases (n)	265,497	34,973	3,585	6,242	4,573	3,638	2,601
	Percentage (% of row)	100.00	13.17	1.35	2.35	1.72	58.28 (% of SU with FEES)	41.67 (% of SU with FEES)
2020	Cases (n)	246,158	33,902	3,475	6,023	4,595	3,604	2,569
	Percentage (% of row)	100.00	13.77	1.41	2.45	1.87	59.84 (% of SU with FEES)	42.65 (% of SU with FEES)
2021	Cases (n)	253,328	35,491	3,710	6,730	5,136	3,976	2,812
	Percentage (% of row)	100.00	14.01	1.46	2.66	2.03	59.08 (% of SU with FEES)	41.78 (% of SU with FEES)
2022	Cases (n)	245,561	32,962	3,466	6,672	5,094	3,944	2,909
	Percentage (% of row)	100.00	13.42	1.41	2.72	2.07	59.11 (% of SU with FEES)	43.60 (% of SU with FEES)
2023	Cases (n)	259,264	34,112	3,450	7,821	5,975	4,575	3,438
	Percentage (% of row)	100.00	13.16	1.33	3.02	2.30	58.50 (% of SU with FEES)	43.96 (% of SU with FEES)
2024	Cases (n)	271,139	34,162	3,238	8,428	6,573	4,965	3,736
	Percentage (% of row)	100.00	12.60	1.19	3.11	2.42	58.91 (% of SU with FEES)	44.33 (% of SU with FEES)
2019–2024	Cases (n)	1,540,947	205,602	20,924	41,916	31,946	24,702	18,065
	Percentage(% of row)	100.00	13.34	1.36	2.72	2.07	58.93 (% of SU with FEES)	43.10 (% of SU with FEES)

FEES, Fiberoptic Endoscopic Evaluation of Swallowing; PEG, Percutaneous Endoscopic Gastrostomy; SU, Stroke Unit

### Analysis stratified by stroke type

**Fig. 2:**
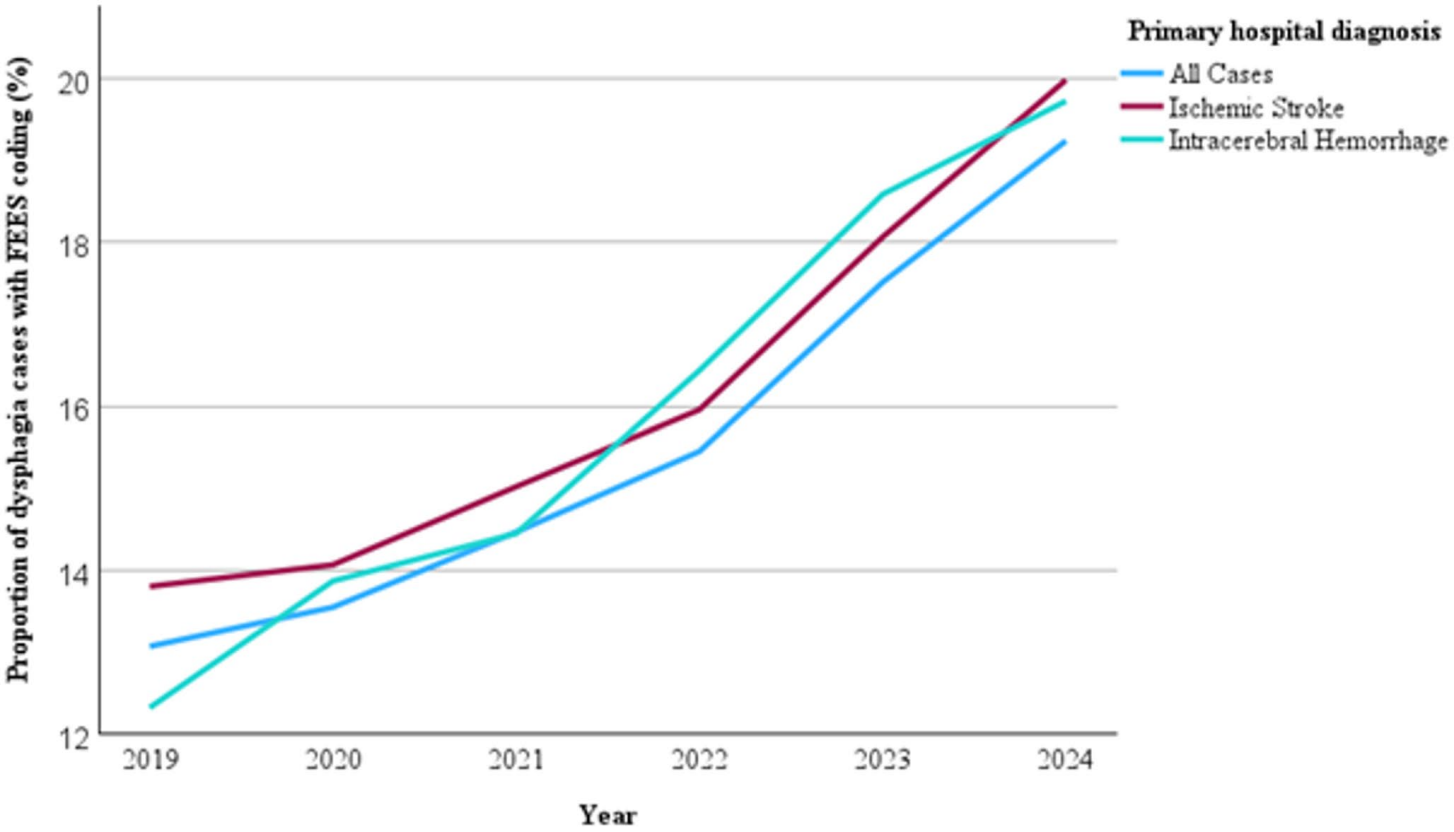
Proportion of cases with FEES and dysphagia coding relative to all dysphagia cases by primary hospital diagnosis over time FEES, Fiberoptic Endoscopic Evaluation of Swallowing

The study cohort comprised 999,227 cases (64.8%) with primary diagnosis of acute ischemic stroke (IS, ICD-10 I63.-) and 71,034 cases (4.6%) with intracerebral hemorrhage (ICH, ICD-10 I61.-), collectively representing 69.5% of all cases with SU care. The remaining cases involved TIA, subarachnoid hemorrhage, or other unspecified stroke diagnoses. When stratifying analyses by primary diagnosis, we identified consistent patterns for both IS and ICH cases (Table 2). Coding for dysphagia was approximately 1.7-fold more frequent in hemorrhagic (28.5%) compared to ischemic cases (16.4%). This difference was even greater for documentation of PEG placement (IS 1.6%; ICH 4.0%). FEES also demonstrated 1.7-fold higher documentation frequency in ICH cases (5.7%) than in IS cases (3.4%) (Fig.1). The proportion of cases with documented FEES amongst all cases with documented dysphagia was broadly comparable between IS (16.1%) and ICH (15.9%) (Fig. 2). Consistent with findings from the overall cohort, cases with male patients predominated among those with a coded FEES examination: IS 58.8% and ICH 62.8%. In our analysis of IS cases, we conducted a subgroup analysis focusing on cases with MT. We found that these cases had higher rates of documentation of FEES (8.6%) and dysphagia (34.1%), which were more than twice those observed in the overall IS case group.

**Table 2:** Documented dysphagia, FEES, and selected case characteristics by primary hospital diagnosis among stroke unit cases, 2019–2024.

	All SU cases	Ischemic Stroke	Intracerebral Hemorrhage	*p*-value*
All SU cases (*n*, % of column)	1,540,947 (100.00)	999,227 (100.00)	71,034 (100.00)	
SU cases with dysphagia (n, % of column)	205,602 (13.34)	163,535 (16.37)	20,262 (28.52)	<0.001
SU cases with PEG placement (n, % of column)	20,924 (1.36)	16,147 (1.62)	2,855 (4.02)	< 0.001
SU cases with FEES (n, % of column)	41,916 (2.72)	34,413 (3.44)	4,075 (5.74)	<0.001
SU cases with both FEES and Dysphagia (n, % of column)	31,946 (2.07)	26,396 (2.64)	3,218 (4.53)	<0.001
SU cases with FEES (male) (n, % of SU cases with FEES)	24,702 (58.93)	20,216 (58.75)	2,559 (62.80)	<0.001
SU cases with FEES (≥ 80years) (n, % of SU cases with FEES)	18,065 (43.10)	15,106 (43.90)	1,450 (35.58)	<0.001

FEES, Fiberoptic Endoscopic Evaluation of Swallowing; PEG, Percutaneous Endoscopic Gastrostomy; SU, Stroke Unit

* Chi-square test comparing proportions between ischemic stroke and intracerebral hemorrhage for each row.

### Analysis stratified by stroke service level

**Fig. 3:**
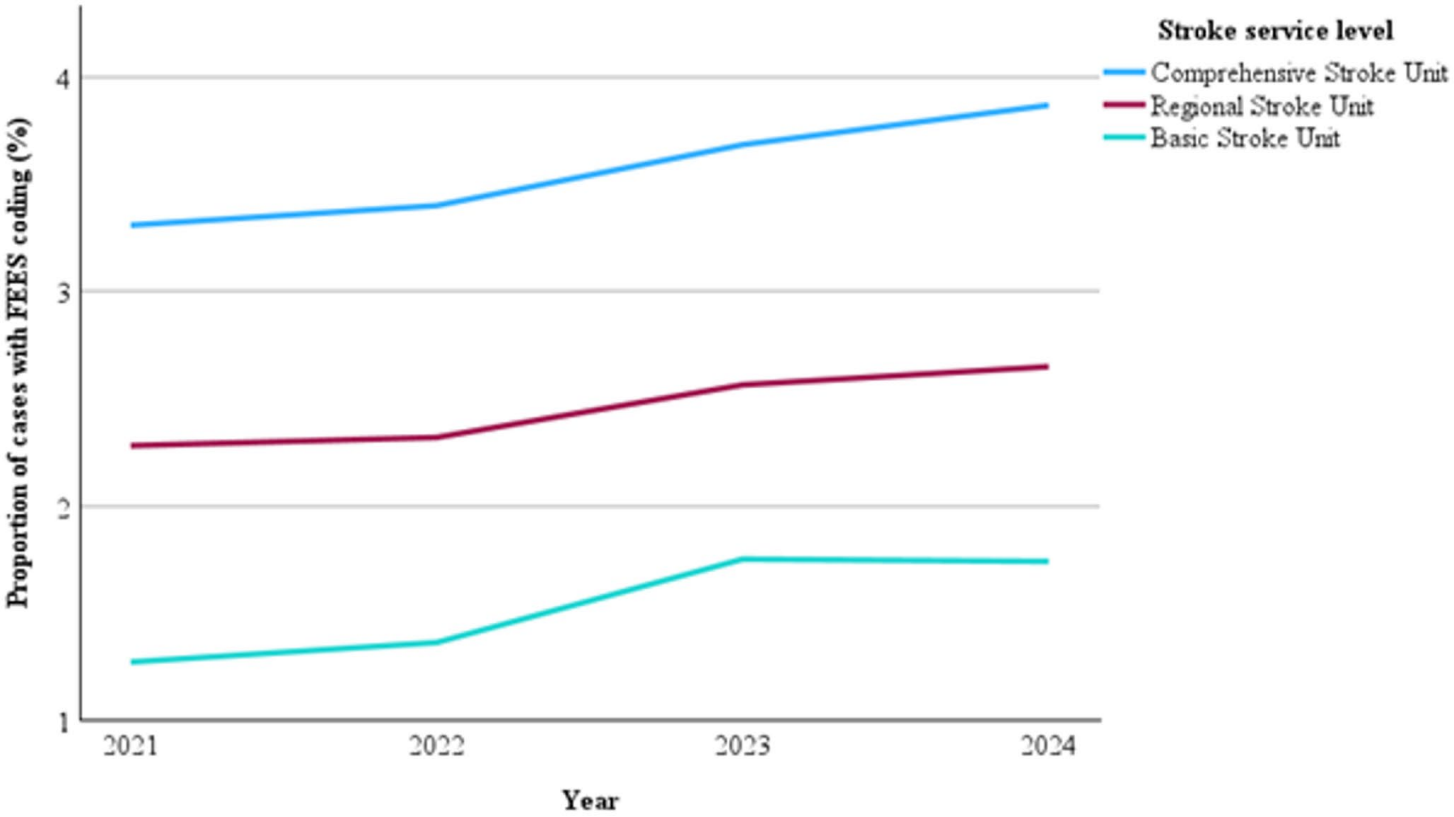
Proportion of cases with FEES coding relative to all SU cases by stroke service level over time FEES, Fiberoptic Endoscopic Evaluation of Swallowing; SU, Stroke unit

**Fig. 4:**
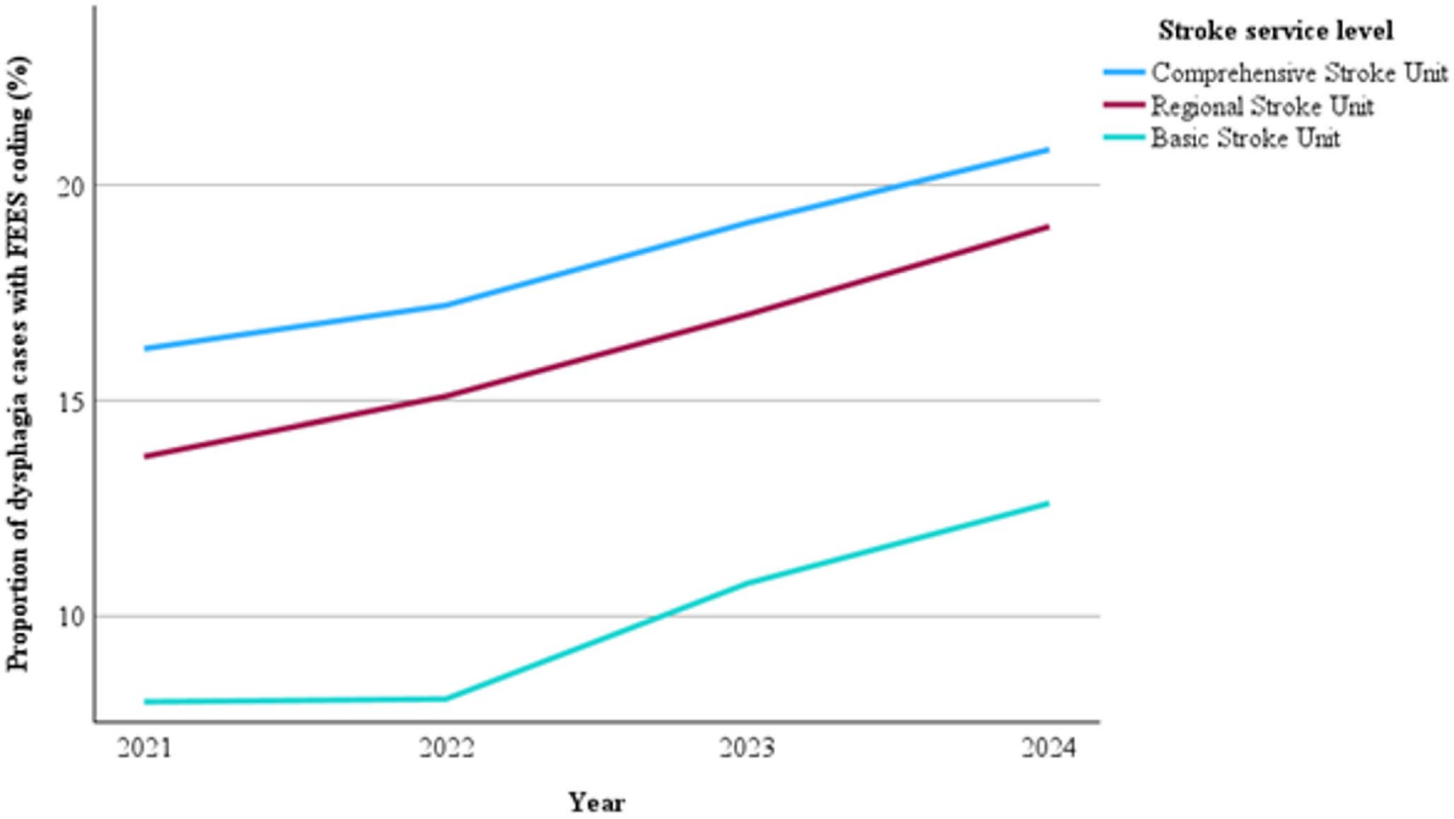
Proportion of cases with FEES and dysphagia coding relative to all dysphagia cases by stroke service level over time FEES, Fiberoptic Endoscopic Evaluation of Swallowing.

For this subgroup analysis, we included cases from 2021 to 2024 (after the OPS code restructuring in 2021) to ensure consistent categorization across the period. The distribution of stroke cases across service levels was as follows: comprehensive stroke units (cSU), 47.5%; regional stroke units (rSU), 41.8%; and basic stroke units (bSU), 10.7%. Coding for dysphagia demonstrated a positive correlation with stroke service level: bSU 11.3%, rSU 12.3%, cSU 14.6%. Similar trends were found for PEG placement: bSU 0.9%, rSU 1.3%, cSU 1.5%. Documentation of FEES followed a parallel pattern: bSU 1.5%, rSU 2.5%, cSU 3.6%, representing a 2.4-fold difference between the highest and lowest service levels (Fig. 3). By 2024, the final year of observation, FEES documentation had reached its maximum across all three service levels (bSU: 1.7%, rSU: 2.7%, cSU: 3.9%), indicating sustained increases at each institutional tier. Similarly, the proportion of cases with FEES coding amongst all cases with dysphagia coding increased with service level: bSU 9.8%, rSU 16.1%, cSU 18.4%, further highlighting the strong correlation between institutional capacity and FEES implementation (Fig. 4). Critically, the temporal trends evident in our primary analysis, specifically the consistent year-to-year increases in FEES documentation and the rising proportion of cases with FEES coding amongst all cases with dysphagia coding, were replicated across all three service levels.

### Sex-specific analysis of FEES utilization and dysphagia rates

We conducted a comprehensive subgroup analysis stratifying all stroke unit cases from 2019 to 2024 by patient sex (Table 3). Males represented 52.6% of total stroke unit cases, while females accounted for 47.4%. Dysphagia documentation was more frequent in females (13.8%) than in males (13.0%); as a result, males represented 51.2% of patients with dysphagia documentation, compared to 52.8% of those without. PEG placement was coded more frequently among cases of male patients (male: 1.5%, female: 1.2%); consequently, males accounted for 58.4% of patients with PEG placement compared to 52.5% of those without. In contrast, cases involving males were coded with FEES more frequently than those involving females (1.29-fold higher): 3.1% versus 2.4%, respectively. While males constituted 58.9% of patients undergoing FEES, they represented only 52.4% of the patients who did not undergo FEES. Most strikingly, when examining the proportion of cases with FEES documentation amongst all cases with dysphagia documentation, a marked sex-based disparity emerged: males 18.1% versus females 12.8%, representing a 1.41-fold difference.

**Table 3:** Documented dysphagia, FEES, and selected case characteristics by sex among stroke unit cases, 2019–2024.*

	All SU cases	Cases with male patients	Cases with female patients	*p*-value**
All SU cases *n*, (% of row)	1,540,947 (100.00)	810,425 (52.59)	730,464 (47.40)	
SU cases with dysphagia (n, % of column)	205,602 (13.34)	105,153 (12.98)	100,440 (13.75)	< 0.001
SU cases with PEG placement (n, % of column)	20,924 (1.36)	12,227 (1.51)	8,696 (1.19)	< 0.001
SU cases with FEES n, (% of column)	41,916 (2.72)	24,702 (3.05)	17,214 (2.36)	<0.001
SU cases with both dysphagia and FEES n, (% of cases with FEES)	31,946 (76.21)	19,041 (77.08)	12,905 (74.97)	<0.001

* Discrepancies in the total number of SU cases and the combined cases of male and female patients resulted from a negligible number of cases with unspecified sex

** Chi-square test comparing proportions between male and female cases for each row.

FEES, Fiberoptic Endoscopic Evaluation of Swallowing; PEG, Percutaneous Endoscopic Gastrostomy; SU, Stroke Unit

## Discussion

### Summary of principal findings

This large-scale, nationwide retrospective analysis of DRG data from the complete six-year observation period (2019–2024) reveals four major and clinically relevant findings regarding FEES and dysphagia documentation patterns in German stroke units:

1. FEES documentation increased consistently in stroke unit cases over the study period, with a stable diagnostic yield throughout all years. However, coding of FEES remains low across all stroke types and institution levels, and increased use may be warranted to align clinical practice with stroke care guidelines.
2. Cases of ICH are coded with dysphagia and PEG placement more frequently than cases of IS, which largely accounts for the higher rates of FEES documentation among ICH cases.
3. A significant sex-based disparity exists in the coding of FEES, which was more frequent in cases with male patients, despite higher dysphagia documentation in cases with female patients.
4. There is a positive correlation between stroke service levels and the documentation of FEES.

### Comparison with existing literature and epidemiological context

#### FEES documentation and adoption trends

We found consistent annual increases in FEES documentation from 2019 to 2024, which aligns well with the established literature on the expansion of FEES adoption across German healthcare systems [14]. This upward trajectory likely reflects multiple converging factors: growing clinical awareness of FEES utility and safety, widespread implementation of the standardized FEES curriculum established in 2014, accumulating evidence supporting the clinical benefit and cost-effectiveness of FEES procedures in selected patient populations, and the recent policy initiatives by the DSG establishing FEES quality benchmarks for stroke unit certification [14, 15, 17]. The publication of multicenter FEES registries demonstrating favorable safety profiles and clinically meaningful therapeutic modifications in the majority of cases has likely contributed to increased clinician confidence in FEES implementation [8, 15, 20]. Our finding that the proportion of cases with documented dysphagia among those coded with FEES remained stable throughout the observation period suggests broadly consistent case selection over time, without evidence of a major shift toward less selective FEES use.

#### Dysphagia and PEG placement documentation patterns

Our overall dysphagia documentation rates were substantially lower than the 50–80% prevalence documented in prospective clinical studies and systematic reviews examining dysphagia following acute stroke [1, 2]. This difference can partly be attributed to the inclusion of cases involving TIA and unspecified strokes in our main analysis. However, our subgroup analysis for ischemic and hemorrhagic strokes also showed dysphagia rates that were lower than those reported in the existing literature. This discrepancy is consistent with findings from other studies that used administrative datasets, which have reported dysphagia rates of 9–10% [11]. Meta analysis which analyzed pooled data from smaller clinical studies reported higher PSD rates [4]. The likely reason for the underestimation of dysphagia is documentation bias in administrative coding systems. Mild, transient dysphagia that resolves quickly is often undocumented, and there may be inconsistencies in documentation standards. In contrast, dysphagia assessments are usually more accurate and thorough in prospective clinical studies. We expected PEG placement among acute stroke patients to be documented more consistently and used it as a surrogate for severe dysphagia. The PEG placement documentation rate was consistent with a previously published rate of 1.9% among acute stroke patients in England from 2007 to 2018 [22]. The relatively low rate of documentation for PEG placements in our study reflects a trend towards more restrictive PEG placement practices in Germany. This trend was highlighted by a recent study of hospital admissions to geriatric units in Germany, which showed a decrease in documented PEG placement among geriatric patients from 1.31% in 2006 to 0.07% in 2024. This study also showed that the proportion of geriatric cases with PEG placement with the main diagnosis of stroke fell from 61.4% in 2006 to 37.0% in 2024 [21]. 

#### Differences in FEES and dysphagia documentation according to stroke subtypes

The substantially higher documentation of dysphagia and PEG placement in our ICH cohort compared to IS patients aligns with published meta-analyses and extensive registry studies characterizing dysphagia epidemiology by stroke subtype [4, 23]. The elevated dysphagia burden in hemorrhagic stroke reflects the characteristic pathophysiology: ICH typically causes more extensive tissue disruption compared to most ischemic events, with greater involvement of motor and sensory cortical and brainstem regions subserving swallowing mechanisms. FEES were coded more frequently in ICH cases. However, the proportion of cases with documented FEES amongst all cases with documented dysphagia was similar for both stroke types, indicating consistent diagnostic decision-making criteria. Thus, the higher FEES documentation rates in ICH cases may be due to a greater dysphagia burden, rather than differences in clinical practice. We focused exclusively on ICH cases treated in SUs. The relatively low percentage of ICH cases in our sample suggests that many more severely affected ICH cases were likely treated in intensive care units and thus were not included in our study. ICH cases treated primarily in intensive care units would probably experience higher rates of dysphagia and FEES procedures due to their more severe clinical condition. Recent findings from a study that conducted FEES screening within five days of admission for patients who underwent MT revealed dysphagia in four out of five patients, which was often overlooked in clinical dysphagia screening tests [2]. Our results confirmed that patients in this subgroup had higher dysphagia documentation rates than in the general IS population, warranting consistent dysphagia screening and greater FEES utilization in this subgroup. The increased rates of dysphagia in this subgroup may be attributed to more severe embolic strokes as well as to dysphagia as a result of intubation.

#### Sex-specific disparities in FEES documentation

Our identification of sex-based disparities in FEES documentation represents a novel and clinically substantial finding warranting discussion and future investigation. Cases with males were coded with FEES substantially more frequently, yet cases with females demonstrated more frequent coding for dysphagia. A higher prevalence of dysphagia has been previously reported among female stroke patients [23, 24]. We found two studies on FEES procedures in neurological patients in Germany that reported similar sex distributions: one evaluated the FEES registry, which included 2,401 patients, of whom 57.8% were male [15]. Another was a cross-sectional hospital-based registry study on 241 consecutive neurological patients who received FEES over three years. In this single-center study, 58.1% of patients who underwent FEES were male [8]. A potential explanation for this disparity can be found in sex-based differences in stroke lesion location, volume, and severity, which may influence dysphagia manifestation and clinical recognition. Male patients are more likely to present with vertebrobasilar lesions that may be preferentially referred for FEES evaluation [25]. We found that coding for PEG placement was also more common in cases involving male patients. This suggests that there were more instances of severe dysphagia among males, which may partly explain the higher documentation of FEES in this group. It has also been previously found that ICH make up a larger proportion of strokes among male stroke patients than among females, which are, in turn, associated with more dysphagia than IS [23, 26]. However, our data do not allow for definitive characterization of the sex-based disparities, but they provide strong evidence of the disparities, warranting further investigation through prospective studies.

#### Institutional variation and service-level effects

Our finding that FEES documentation rates increase with stroke service level reflects well-documented patterns in healthcare delivery capacity and quality of care [27]. While our findings indicate that the trend can be partly linked to an increase in the documentation of both dysphagia and PEG placement as the stroke service level rises, the steady rise in the proportion of FEES documentation among all cases with documented dysphagia suggests that institutional factors may affect case selection and the capacity for conducting FEES procedures. This finding should be taken into account in future adaptations of DSG quality benchmarks for the certification of SUs. To reduce the variability in FEES procedures between hospitals and stroke service levels, it is important to address several key issues. These include staffing requirements, access to standardized FEES training, and the integration of workflows to create clear referral pathways and protocols. One solution may involve developing standardized criteria for the use of FEES following an acute stroke. Furthermore, gradually integrating quality benchmarks into SU certification criteria and national treatment guidelines could help ensure consistency in treatment.

#### Strengths of this analysis

This analysis represents the largest and most comprehensive assessment of FEES implementation in German SUs to date. This large sample size provides robust statistical power for all subgroup analyses. Our study provides complete nationwide coverage of all acute-care hospitals across Germany. This eliminates selection bias inherent to survey-based studies or analyses restricted to specific hospital networks. Consistent temporal trends across yearly, cumulative, and all stratified subgroup analyses (by stroke type, service level, and patient sex) substantially strengthen the robustness and generalizability of our findings.

#### Limitations of this analysis

Several limitations must be acknowledged: First, administrative data depend on the accuracy and completeness of medical coding. Dysphagia documentation as a secondary diagnosis, ICD-10 code R13.-, likely underestimates true dysphagia prevalence, particularly for mild, transient dysphagia or dysphagia not formally assessed. This documentation bias limits the sensitivity of dysphagia prevalence estimates derived from administrative data. Importantly, our analyses reflect coded procedures and coded diagnoses in administrative data rather than the true underlying number of procedures performed or cases affected; accordingly, reported frequencies may differ from real-world procedure and case counts because of undercoding, miscoding, or other coding-related biases. Furthermore, administrative coding does not capture the temporal sequence or clinical context of assessments and procedures. We therefore could not determine whether dysphagia was identified before or after FEES, whether FEES was performed for diagnostic confirmation or therapy guidance, or whether uncoded bedside assessments influenced procedural use. Second, procedures for FEES may be less consistently documented in administrative records than SU treatment codes, because coding for FEES procedures does not by itself yield higher reimbursement. We also cannot rule out the possibility that a limited number of cases may have received multiple OPS codes for SU treatment in our subgroup analysis categorized by stroke service level; however, we expect this number to be negligible. Third, administrative data do not provide information regarding stroke severity, lesion characteristics, detailed clinical presentation features, or other clinical variables that might explain sex-based disparities in FEES utilization. Fourth, we only reported data on cases with coding for stroke unit treatment, which may limit the generalizability of our findings to the broader stroke patient population in Germany, particularly for those treated solely in intensive care units who tend to be more severely affected. Finally, because this was a retrospective observational analysis based on administrative data, causal relationships cannot be established. The observed differences by stroke service level, stroke subtype, and sex may be influenced by unmeasured confounders such as stroke severity, lesion location, comorbidity burden, local workflow structures, or hospital-specific documentation practices. Prospective studies specifically designed to investigate mechanisms underlying sex-based disparities and service-level variations would provide valuable complementary evidence.

## Conclusion

This nationwide analysis provides baseline data on the implementation of Fiberoptic Endoscopic Evaluation of Swallowing (FEES) in German stroke units from 2019 to 2024. Although documentation of FEES has increased annually, it remains substantially below dysphagia documentation rates, indicating areas for improvement. Our findings suggest that there are disparities in FEES documentation based on sex, which warrant further investigation. Additionally, institutional factors influence the capacity for FEES procedures, underscoring the need for differentiated benchmarks for stroke unit certification criteria. Improvements in reimbursement policies, along with support for staff training and streamlined workflows, could enhance access to FEES for stroke patients in Germany.

## Data Availability

Data will be made available by the corresponding author upon reasonable request.

## Abbreviations

DRG: Diagnosis-related group.
DSG: German Stroke Society (Deutsche Schlaganfall-Gesellschaft)
FEES: Fiberoptic endoscopic evaluation of swallowing.
ICH: Intracerebral hemorrhage.
InEK: Institut für das Entgeltsystem im Krankenhaus (Institute for the Hospital Remuneration System).
IS: Ischemic stroke.
MT: Mechanical thrombectomy.
OPS: Operationen- und Prozedurenschlüssel (German operation and procedure classification)
PEG: Percutaneous endoscopic gastrostomy.
PSD: Post-stroke dysphagia.
SU: Stroke unit.
TIA: Transient ischemic attack
